# *Anopheles gambiae *distribution and insecticide resistance in the cities of Douala and Yaoundé (Cameroon): influence of urban agriculture and pollution

**DOI:** 10.1186/1475-2875-10-154

**Published:** 2011-06-08

**Authors:** Christophe Antonio-Nkondjio, Billy Tene Fossog, Cyrille Ndo, Benjamin Menze Djantio, Serge Zebaze Togouet, Parfait Awono-Ambene, Carlo Costantini, Charles S Wondji, Hilary Ranson

**Affiliations:** 1 Laboratoire de Recherche sur le Paludisme, Organisation de Coordination pour la lutte Contre les Endémies en Afrique Centrale (OCEAC), P.O. Box 288, Yaoundé, Cameroon; 2 Faculty of Sciences, University of Yaoundé I, P.O. Box 337, Yaoundé, Cameroon; 3 Institut de Recherche pour le Développement (IRD), UR 016, 911, avenue Agropolis, P.O. Box 64501, 34394 Montpellier cedex 5, France; 4 Vector group, Liverpool School of Tropical Medicine, Pembroke Place, Liverpool L3 5QA, UK

## Abstract

**Background:**

Urban malaria is becoming a major health priority across Africa. A study was undertaken to assess the importance of urban pollution and agriculture practice on the distribution and susceptibility to insecticide of malaria vectors in the two main cities in Cameroon.

**Methods:**

Anopheline larval breeding sites were surveyed and water samples analysed monthly from October 2009 to December 2010. Parameters analysed included turbidity, pH, temperature, conductivity, sulfates, phosphates, nitrates, nitrites, ammonia, aluminium, alkalinity, iron, potassium, manganese, magnesium, magnesium hardness and total hardness. Characteristics of water bodies in urban areas were compared to rural areas and between urban sites. The level of susceptibility of *Anopheles gambiae *to 4% DDT, 0.75% permethrin, 0.05% deltamethrin, 0.1% bendiocarb and 5% malathion were compared between mosquitoes collected from polluted, non polluted and cultivated areas.

**Results:**

A total of 1,546 breeding sites, 690 in Yaoundé and 856 in Douala, were sampled in the course of the study. Almost all measured parameters had a concentration of 2- to 100-fold higher in urban compare to rural breeding sites. No resistance to malathion was detected, but bendiocarb resistance was present in Yaounde. Very low mortality rates were observed following DDT or permethrin exposure, associated with high *kdr *frequencies. Mosquitoes collected in cultivated areas, exhibited the highest resistant levels. There was little difference in insecticide resistance or *kdr *allele frequency in mosquitoes collected from polluted versus non-polluted sites.

**Conclusion:**

The data confirm high selection pressure on mosquitoes originating from urban areas and suggest urban agriculture rather than pollution as the major factor driving resistance to insecticide.

## Background

Malaria represents a major threat for human population development across Africa [1]. Until recently, urban development was generally believed to reduce the risk of vector breeding sites and thus malaria transmission [2]. However, millions of clinical episodes of malaria occur annually in urban areas, indicating that the epidemiology of this disease is changing [3]. In Cameroon, it is estimated that over 52% of the population live in urban areas and the total urban population has almost doubled in the last 25 years [4]. This process has been accelerated by economic crises over the past 20 years, which had the biggest impact on rural dwellers leading many people to migrate from rural to urban areas.

The two cities of Douala and Yaoundé have experienced the highest demographic growth. Estimated to be less than 500 000 inhabitants in the mid 1970s, the population is now over 2.5 million inhabitants in each city [4]. This urban population growth has outpaced urban planning and sanitation infrastructures and, this rapid spontaneous urbanization in and around city centres is typically characterized by poor housing, the absence of any urban plan in crowded districts, lack of sanitation and drainage of surface water, and the colonization of lowlands for urban agriculture or for house construction. As a result, an increasing number of districts are victims of flooding during rainy seasons. The situation has led to increased mosquito densities and greater risk of several vector borne diseases, such as malaria, dengue or chikungunya [5-8]. Although malaria transmission rates still remain higher in the suburban areas compared to central districts, clinical malaria attacks and severe episodes are frequent in both areas [9,6,10]. For over a decade now, major malaria control efforts in the country have been directed toward adult mosquito control including use of impregnated bed nets, screens, insecticide sprays and coils [11,12]. The national malaria control programme has distributed over two millions impregnated bed nets since 2000, mainly to urban dwellers [10]. However, this has had a limited impact on the disease burden. This may be partially due to insecticide resistance in the malaria vectors, which has increased across the country [13,12], but probably also reflects the insufficient assessment of local risks factors. There is a clear need for a thorough assessment of malaria transmission risks across ecological foci, and an evaluation of the efficacy of control measures targeting the aquatic stages of mosquitoes which could constitute an important component of the vector control strategy in urban areas as it is already the case in several African cities [14-17]. In Douala and Yaoundé, where there is a relatively easy access to almost all major breeding habitats, larval control could be cost-effective and sustainable. In the present study, the distribution of malaria vectors breeding sites in the two cities was assessed and the characteristics of rural and urban breeding sites compared. Breeding sites in urban settings were classified as originating from cultivated areas, polluted and non-polluted sites and the vector susceptibility to insecticides was assessed in each type of site.

## Methods

### Study sites

The study took place in the cities of Yaoundé (3° 51'N 11° 30'E) and Douala (3° 48'N 10° 08'E) the two major urban cities in Cameroon. These cities are situated within the Congo-Guinean phytogeographic zone characterized by a typical equatorial climate with two rainy seasons extending from March to June and from September to November. Douala is situated near the Atlantic coast 1 m above sea level and receives over 3,500 mm of rainfall annually whereas the annual average rainfall in Yaoundé is 1,700 mm. Yaoundé is located inland 250 km east of Douala. The city is situated 800 m above sea level and is surrounded by many hills.

The study was conducted under the ethical clearance N° 216/CNE/SE/09 delivered by the Cameroon National Ethics Committee Ref N° IORG0006538-IRB00007847-FWA00016054.

**Figure 1:**
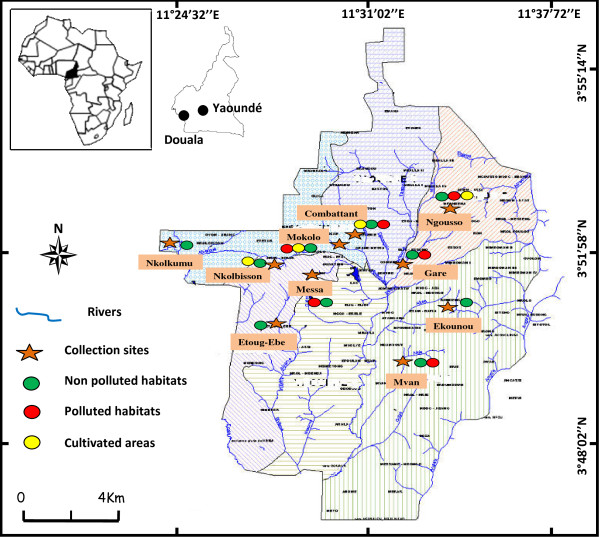
**A map of the city of Yaoundé showing the location of collection sites and the various type of breeding site with anopheline frequently found in the area**.

**Figure 2:**
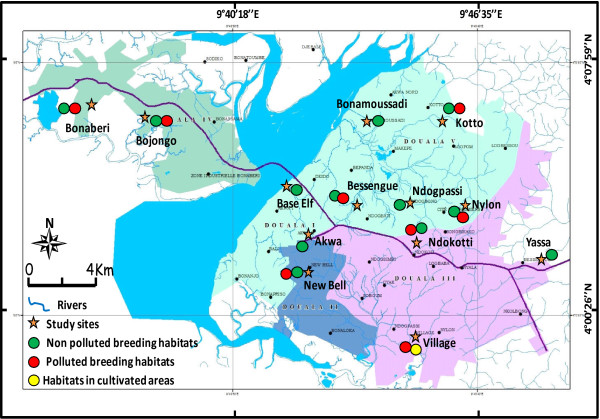
**A map of the city of Douala showing the location of collection sites and the type of breeding site with anopheline frequently found in the area**.

Field collections in the city of Yaoundé were carried out in ten districts: Mokolo, Messa, Etoug-Ebe, Combattant for densely populated and/or commercial areas, Ngousso, Nkolmessen, Ekounou, Mvan for residential areas with low density population, Nkolbisson (suburban area), and Nkol kumu in the rural area (Figure 1). In Douala, field collections took place in thirteen districts: Bonaberi, Bessengue, Base Elf, Akwa, Nylon, Ndokoti situated in the industrial and/or commercial zone, Bojongo, Bonamoussadi, Kotto, New-Bell, Ndog-passi, village, located in densely populated residential districts and Yassa in the suburban area (Figure 2). In both cities built up areas are interspersed with open sparsely populated areas. Areas with high rise buildings or established residential areas are surrounded by temporary or 'shanty' urbanized areas. In Yaoundé the established urban sites are constructed on the hills slopes with low land areas used for temporary/lower quality habitat construction in densely populated districts or exploited for urban agriculture.

### Larval collection and habitat characterization

Mosquito larval collections to assess anopheline distribution in the cities of Douala and Yaoundé were conducted once every three months from October 2009 to December 2010. following WHO protocols [18]. All open water bodies were taken as potential breeding sites and explored. Presence or absence of larvae was determined after 10 to 15 dips. Arbitrarily, sites with over 200 larvae were considered having high larval density. Sites with less than 50 larvae were considered as low-density habitats. Sites with no anopheline larvae were recorded as absent. The presence of culicine larvae alongside anopheline larvae was systematically recorded. The breeding sites were categorized as follows: swamps, drains, pools, puddle or ditches, footprints, tyre track, artificial containers and others. Physical characteristics of the open water collection sites were recorded by the same person, in order to maintain consistency in visual classifications. Further parameters noted were the general surrounding (presence or absence of high density houses), distance to the nearest house, human activities related with the creation of breeding sites (house construction, urban farming, colonization of swamps etc) and the presence or absence of predators. The proportion of the water surface covered by the vegetation was estimated visually and the type of vegetation recorded as grass, water lentils, and algae. The identification of predators was limited to the presence of larvivorous fishes.

Measurements of physicochemical characteristics of breeding sites were recorded using a Wagtech portable Kit (CP1000). Parameters measured were: turbidity expressed in Nephelometric Turbidity Unit (NTU), pH, temperature (°C), conductivity expressed in micro Siemens per cm (μs/cm), sulfates, phosphates, nitrates (NO-3), nitrites (NO-2), ammonia (NH+4), aluminium, alkalinity, iron, potassium, manganese, magnesium; Magnesium hardness and total hardness concentrations all expressed in mg/l. Breeding places were classified in three categories after visual inspections: Polluted breeding sites, Non polluted breeding sites, Cultivated breeding sites.

-"Polluted breeding sites" are semi permanent water collections containing domestic wastes, or organic product in decomposition which could be invaded by moisture or alga.

-"Non polluted breeding sites" are temporary water collections created after rains or resulting from a clean water source and mainly without any sign of organic pollution.

-"Cultivated breeding sites" are created by the practice of agriculture and include furrows and irrigation pits

Rural breeding sites were temporary water collections sampled in rural areas situated 12 to 15 km away from the city centre.

### Mosquito identification

Anopheline larvae were identified morphologically using the Gillies and Coetzee keys [19]. Culicinae were identified morphologically following the Edwards keys [20]. Mosquitoes belonging to the *Anopheles gambiae *complex were subjected to PCR assays designed for species and molecular forms identifications [21]. Genomic DNA used for molecular analysis was extracted from dessicated adult mosquitoes according to either Livak [22] or Cornel [23] protocols.

### Insecticide susceptibility assays

Bioassays were carried out using WHO test kits for adult mosquitoes [24]. The following diagnostic concentrations of insecticides were tested: 4% DDT, 0.75% permethrin, 0.05% deltamethrin, 0.1% bendiocarb and 5% malathion.

Susceptibility tests were carried out with 2 to 4 day- old unfed *An. gambiae *s.l. adults raised from larvae collected in breeding sites. Batches of 20 to 25 mosquitoes per tube were exposed to impregnated papers for one hour. The number of mosquito knockdown was recorded every 5 minutes during exposure. After exposure, mosquitoes were supplied with glucose solution as food, and mortality was recorded 24 hours post-exposure. Tests with untreated papers were systematically run as controls. Mortality rate in tested samples was corrected using Abbot formula [25] when the mortality rate of control was between 5-20%. WHO criteria [24] were used to evaluate the resistance and susceptibility status of the tested mosquito population. The resistance status was indicated by a mortality rate below 80% while mortality rates greater than 97% were indicative of susceptibility and mortality rates between 80 - 97% suggest increased tolerance, but resistance should be confirmed.

To screen for the presence of the *kdr *alleles (L1014F and L1014S) conferring resistance to DDT and pyrethroids, DNA extracted from individuals exposed to insecticide was tested using the Hot Ligation Oligonucleotide Assay (HOLA) of Lynd *et al *[26] and the TaqMan assay [27]. A subset of mosquitoes exposed to bendiocarb were screened for the presence of the Acetylcholinesterase 1 target-site resistance mutation G119S (ace-1^R^) using the TaqMan assay [27].

### Statistical analysis

Prior to the analysis, all physical characteristics of breeding sites were transformed into categorical variables. The relationships between the presence or absence of immature stages of anopheline and potential explanatory variables (measured end categorical variables) were first tested with univariate analysis. *P*-values < 0.05 were considered significant. Then the presence or absence of immature stages was analysed using logistic binary regression with a conditional stepwise procedure. All variables significantly associated with the dependent variable in univariate analysis, and variables with *P*-values < 0.25, were introduced into the model. The goodness of fit of the final model was assessed using the Hosmer and Lemeshow statistic. All these analysis were performed using SPSS V 12.0. Percentages were compared using Pearson's chi squared test or Fisher's exact test. Comparison between means was assessed using ANOVA or Kruskals Wallis test in case of inequality of sample variances. To compare the characteristics of breeding sites between 1) the cities of Douala and Yaoundé and 2) categories of breeding habitats, all measured physicochemical variables were transformed into principal component (independent variables). Then a logistic regression analysis was applied to compare the distribution of the breeding habitats in each city according to these new predictors. A linear discriminant function analysis was used to determine key factors associated with the classification of breeding sites into polluted, non-polluted and cultivated areas. These analyses were performed using the software R and EPI-INFO V3.5.2.

For bioassay analyses, comparison of percentages was performed using the chi square test. Estimates of mortality rates and the relationship between phenotypes and genotypes were carried using the software MedCalc V11.5.0.0. Genotypes frequencies at the *kdr *locus were tested against Hardy Weinberg equilibrium for each city and for each category of breeding habitats. Statistical significance was assessed by the exact probability test using GENEPOP V4. [28].

## Results

### Larval identification and distribution

A description of the general characteristics of mosquito larval habitats in the two cities is presented in Table 1. A total of 1,546 breeding sites corresponding to 690 breeding sites in Yaoundé and 856 in Douala were sampled in the course of the study. 534 sites were found with anopheline larvae. *Anopheles gambiae *s.s. was the only anopheline species collected in both sites. Of the 495 Douala specimens successfully identified to molecular forms, 494 were of the M molecular form and one of the S molecular form. In Yaoundé, out of the 382 specimens analysed 379 were of the M molecular form and 3 of the S molecular form. Culicine larvae were mainly composed of *Culex *(*Culex duttoni, Culex tigripes, Culex quinquefasciatus*), *Aedes aegyti *and *Aedes albopictus*. Despite seasonal variations, available breeding places with anopheline larvae remained important all year round varying from 24% positive sites during the long rainy season to 65% during the short dry season. The persistence of breeding sites may be attributed to the particularly long rainy season in the equatorial forest region and human activities such as cultivation of lowlands, washing of cars, and domestic activities. The association between anopheline and culex in the same site was found to be important particularly during the long dry season when the proportion of available breeding opportunities decreased (Table 1).

**Table 1:** Characteristics of breeding sites sampled in the cities of Douala and Yaoundé from October 2009 to December 2010

	Number of Breeding sites sampled	BS with anopheline	BS with culex & anopheline	% BS with high larval density	% BS with larvivorous fishes	% BS less 3 m²	% BS < 10 m from the nearest inhabited house	% BS with vegetation	>70% of BS surface exposed to sunshine
**Douala**									
***Long rainy season ***(oct-nov 2009)	416	102 (24.5%)	12 (3%)	57.1%	0%	72.4%	73.7%	15.8%	67.7%
***Long dry season*** (feb - mar 2010)	100	29 (29%)	24 (24%)	2.6%	10.1%	51.3%	68.9%	47.9%	71.4%
***Short rainy season ***(may - jun 2010)	126	79 (63%)	28 (22.2%)	20.8%	4.2%	59.2%	60%	45.8%	85.2%
***Short dry season ***(aug-sept 2010)	146	95 (65%)	27 (18.5%)	16.8%	4.8%	40.4%	56.6%	37.7%	95.6%
***Long dry season*** (december 2010)	68	33 (48.5%)	18 (47.4%)	7.9%	10.7%	47.6%	9.5%	39.3%	79.8%
**Yaoundé**									
***Long rainy season ***(oct-nov 2009)	201	58 (28.9%)	18 (9%)	40%	2.4%	68.3%	41.4%	32.4%	77.4%
***Long dry season*** (feb - mar 2010)	115	40 (34.8%)	34 (29.6%)	0%	15%	57.5%	23%	45.1%	92.9%
***Short rainy season ***(may - jun 2010)	173	81 (46.8%)	26 (15%)	12.3%	12%	45.7%	11%	57.2%	91.9%
***Short dry season ***(aug-sept 2010)	117	40 (34%)	13 (11.1%)	5%	0.8%	70.9%	24.8%	9.4%	88.4%
***Long dry season*** (december 2010)	84	38 (45.2%)	14 (42.4%)	9%	11.8%	47%	47%	32.3%	66.2%

BS: Breeding Sites

Of the 534 sites productive for anopheline larvae, 53% and 66% had low larval densities in Douala and Yaoundé respectively. Large drains and pools did contribute to the anopheline burden, but permanent breeding places, which could constitute dry season refuge for anopheline, appeared less productive than temporary breeding sites. This may reflect the presence of numerous predator or competitors in the larger sites or may be a bias as resulting from difficulties in sampling fully in these sites due to their large sizes and the presence of the aquatic vegetation.

### Physicochemical characteristics of breeding sites

#### Comparison of water quality between urban and rural breeding sites

Breeding sites from urban areas were compared to rural breeding sites to assess the influence of urbanization on mosquito breeding environment in the two cities. Almost all measured physicochemical parameters had values 2- to 100-fold higher in urban compared to rural breeding sites. However, with the exception of turbidity, potassium and ammonia in Douala and total hardness and conductivity in Yaoundé most of the parameters were not significantly different in urban and rural areas (Table 2). This could result from the low statistical power of the test due to the low sample size in rural areas or to the absence of mark differences between urban and rural areas for the parameters considered.

**Table 2:** Comparision of physico chemical characteristics of breeding sites from urban and rural areas in Douala and Yaoundé.

	Douala			Yaoundé		
	
	Rural breeding sites	Urban breeding sites	P value	Rural breeding sites	Urban breeding sites	P value
N	6	109		7	158	
Alcalinity	105.8 ± 44.7	113.5 ± 2.04	0.63	105.7 ± 30.7	173.2 ± 1.9	0.22
Aluminium	0.13 ± 0.07	0.68 ± 0.08	0.81	0.05 ± 0.03	0.028 ± 0.001	0.97
Ammonia	0.14 ± 0.05	1.1 ± 0.03	**0.005**	0.71 ± 0.41	2 ± 0.03	0.20
Nitrates	1.53 ± 0.5	5.38 ± 0.15	0.105	1.43 ± 0.4	3.37 ± 0.05	0.19
Nitrites	0.01 ± 0.006	0.28 ± 0.02	0.19	0.065 ± 0.02	0.28 ± 0.007	0.25
Manganese	0.012 ± 0.004	31.5 ± 1.5	0.105	0.008 ± 0.002	0.01 ± 0.0002	0.93
Potassium	4.55 ± 0.8	12.3 ± 0.8	**0.016**	12.17 ± 5.01	12.7 ± 0.58	0.90
Iron	5.53 ± 0.61	13.8 ± 0.9	0.24	2 ± 1.25	4.2 ± 0.13	0.08
Phosphate	0.5 ± 0.18	0.76 ± 0.04	0.39	0.65 ± 0.18	0.93 ± 0.01	0.71
Sulfate	10.5 ± 4.9	29.15 ± 2.7	0.61	13.9 ± 4	34 ± 0.5	0.34
Mg hardness	1.67 ± 1.3	17.3 ± 0.3	0.23	6.07 ± 4.5	19.8 ± 0.5	0.12
Total hardness	52.5 ± 18.9	57.3 ± 1.1	0.90	27.1 ± 8.8	111.1 ± 1.19	**0.003**
Conductivity	205.57 ± 37.9	393.4 ± 3.6	0.11	122.7 ± 37.6	830.8 ± 11.2	**0.000**
Turbidity	177.5 ± 20	105.2 ± 2.6	**0.006**	89.2 ± 38.2	103.7 ± 1.9	0.61
pH	6.84 ± 0.26	6.63 ± 0.01	0.76	5.9 ± 0.6	6.8 ± 0.016	0.55
Dissoved Oxygen	115.3 ± 7.8	170.8 ± 25.5	0.61	50.7 ± 21.5	94.2 ± 1.6	0.09

Significant if P < 0.05

#### Comparison between Douala and Yaoundé

**Figure 3:**
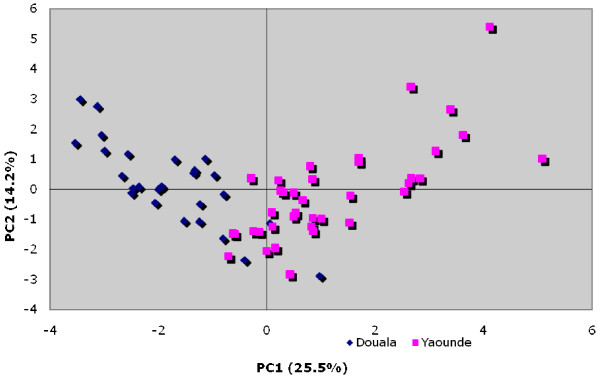
**A principal component regression analysis comparing physico-chemical characteristics of breeding sites in both Douala and Yaoundé**.

To assess whether breeding sites in Yaoundé and Douala differed, in their physicochemical composition, all measured parameters were transformed to independent variables (principal components) due to the high correlation between environmental variables. Logistic regression analysis demonstrated that the larval habitats in Douala and Yaoundé had very different physicochemical characteristics. The ordination diagram constructed with the first two axis explaining about 40% of the total variance, show a non ambiguous difference between breeding sites of the two cities (Figure 3). The first axis was found to be positively correlated with nitrites, total hardness, phosphate, ammonium, alkalinity and conductivity while, the second axis was positively correlated with nitrites, magnesium hardness, aluminium, ammonium, nitrates, manganese, alkalinity, sulfate, conductivity and temperature.

#### Comparison of mosquito breeding habitats physicochemical characteristics in urban areas

To compare the natural breeding environment for mosquitoes in urban areas and assess differences between sites, mosquito breeding habitats in Douala and Yaoundé, were separated into three different categories after visual inspections: polluted, non polluted and cultivated areas. Again, few significant differences were observed (P < 0.05), conductivity, turbidity, phosphate and potassium in Douala and conductivity, aluminium, phosphate and sulfate in Yaoundé. Conductivity in both cities, turbidity and potassium in Douala were found to be more important in polluted sites. Phosphates level was higher in cultivated areas (Table 3).

**Table 3:** Average values of physico-chemical parameters of cultivated, polluted and non polluted sites sampled in Douala and Yaoundé from January to December 2010.

	Douala				Yaoundé			
	
	Cultivated areas	Non Polluted	Polluted	P value	Cultivated areas	Non Polluted	Polluted	P value
N	12	68	50		53	54	41	
Alcalinity	35.3 ± 11.1	117.3 ± 3.8	125.2 ± 5.84	0.1	136.9 ± 5.6	184.7 ± 5	201.1 ± 6.9	0.09
Conductivity	192.4 ± 24.8	294.9 ± 3.7	505.7 ± 9.6	**0.000**	608.8 ± 28.4	705.8 ± 24.2	1188.3 ± 44.8	**0.005**
Turbidity	127 ± 38.1	73.4 ± 4.2	146 ± 7.7	**0.000**	93.98 ± 5	78.2 ± 3.9	148.2 ± 8.4	0.05
pH	5.89 ± 0.2	6.61 ± 0.02	6.77 ± 0.12	0.12	6.46 ± 0.05	7.04 ± 0.04	6.96 ± 0.05	0.07
Temperature	29.9 ± 0.4	29.6 ± 0.06	30.2 ± 0.1	0.60	28.69 ± 0.18	27.93 ± 0.1	27.7 ± 0.13	0.30
Ammonia	1.03 ± 0.4	1.02 ± 0.05	1.03 ± 0.07	0.06	1.84 ± 0.1	1.83 ± 0.09	2.4 ± 0.1	0.4
Nitrates	4.8 ± 1.2	4.01 ± 0.2	7.42 ± 0.5	0.29	2.7 ± 0.09	3,53 ± 0.18	4.02 ± 0.21	0.28
Nitrites	0.05 ± 0.01	0.5 ± 0.05	0.07 ± 0.007	0.68	0.26 ± 0.02	0.22 ± 0.01	0.37 ± 0.03	0.49
Aluminium	0.96 ± 0.3	0.124 ± 0.01	1.4 ± 0.14	0.08	0.01 ± 0.0006	0.045 ± 0.003	0.032 ± 0.004	**0.03**
Manganese	73.5 ± 27	6.8 ± 1.6	55.2 ± 4.8	0.19	0.012 ± 0.0007	0.013 ± 0.0005	0.007 ± 0.0004	0.14
Phosphate	0.99 ± 0.3	0.67 ± 0.05	0.69 ± 0.14	**0.025**	1.12 ± 0.04	0.60 ± 0.075	1.1 ± 0.05	**0.03**
Sulfate	27.7 ± 5.7	24.8 ± 1.03	36.8 ± 2	0.67	16.3 ± 1	45.78 ± 1.4	38.4 ± 2.2	**0.001**
Mg Hardness	10.2 ± 2.6	24.3 ± 1.6	9.4 ± 1.03	0.06	16 ± 1.9	24.07 ± 3.4	21.9 ± 3	0.46
Total hardness	20 ± 4.5	57.4 ± 1.9	60.6 ± 3.4	0.37	94 ± 3.5	116.7 ± 3.6	125.6 ± 3.8	0.06
Magnesium	1.67 ± 1.9	3.04 ± 0.56	0.6 ± 0.3	0.18	22.4 ± 2.4	23.3 ± 1.7	24.7 ± 2	0.97
Iron	-	12.9 ± 1.4	23.2 ± 6.4	0.16	3.2 ± 0.3	5.4 ± 0.4	3.8 ± 0.4	0.11
Potassium	-	8.6 ± 0.69	21.5 ± 3.5	**0.03**	13.4 ± 2.4	7.8 ± 0.6	18.3 ± 2.2	0.05
Dissolve oxygen	-	89.9 ± 6.5	360.9 ± 309	0.31	122.9 ± 9.2	85.8 ± 2.8	84.3 ± 0.9	0.17

Significant if P < 0.05

**Figure 4:**
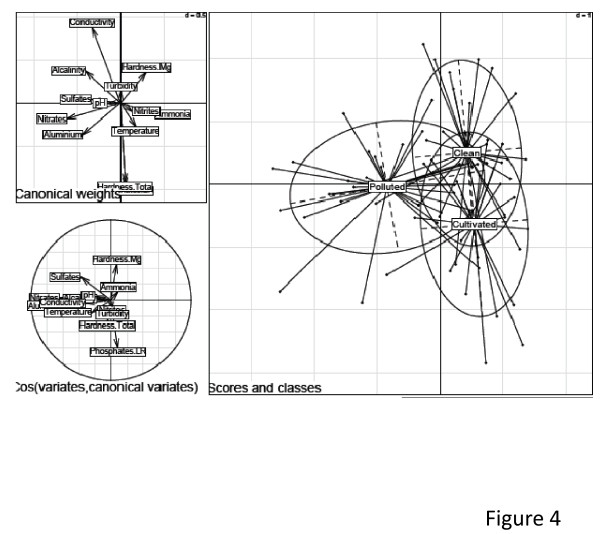
**Linear Discriminant Analysis (LDA) diagrams showing the correlation between the classification of breeding sites in polluted, non polluted and cultivated areas and physico chemical characteristics**. The first axis is horizontal, second vertical (Non polluted = clean). The figure at the top shows the canonical weight of each variable the size of arrows indicate the importance of the parameter.

A Linear Discriminant Analysis (LDA) was then performed to identify variables maximally discriminating each category of habitats. The data from both cities were combined to increase the power of the analysis. The resulting diagram draw with the first two axes explaining about 40% of the total variance presented the following parameters: conductivity, total hardness, nitrates, aluminium, alcalinity, phosphate, magnesium hardness, sulfate as the major parameters discriminating the three categories of breeding sites (Figure 4).

#### Association between anopheline larvae presence and physicochemical parameters

A binary logistic regression model was used to test the association between the presence of *An. gambiae *immature stages and characteristics of the breeding site. The analysis of the pooled sample of both cities showed three parameters: the presence of organic pollutants in breeding sites, presence/absence of Culex, and the size of breeding sites to be significantly associated with *An. gambiae *immature stage presence. The presence of Culex was positively correlated to the presence of anopheline larvae (RR = 2.97; 95% CI 1.57-5.41). In the other hand, the presence of organic pollutants in breeding habitats (RR = 0.561; 95% CI 0.38-0.83) and the large size of breeding sites (RR = 0.485; 95% CI 0.26-0.89) were negatively correlated to the presence of anopheline larvae.

### Susceptibility to insecticides

**Figure 5:**
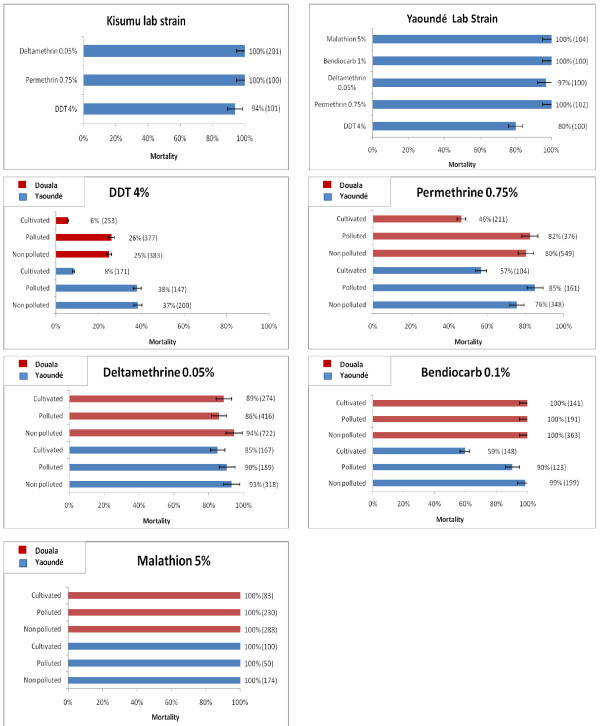
**Diagrams showing the level of susceptibility of *An. gambiae *adults collected in different type of breeding sites after one hour exposition to insecticides**.

A total of 7,656 *An. gambiae *females (2599 in Yaoundé and 5057 in Douala) aged 2-4 days were tested for susceptbility to insecticides. The Kisumu *An. gambiae *susceptible strain and the OCEAC lab strain (Yaoundé strain) were used as controls. In all sites, 100% mortality after exposure to malathion was observed. Similarly complete mortality was observed after exposure of the Douala mosquitoes to bendiocarb irrespective of their breeding site origin. However, in Yaoundé, while high mortality was observed for the non polluted sites (99%) and polluted sites (90%) only 55% of adults raised from larvae collected in cultivated areas were killed by the discriminating dose of bendiocarb (Figure 5).

There was a very low mortality with DDT (4%) particularly in cultivated areas where a mortality rate of 6 and 8% was recorded in Douala and Yaoundé respectively. Mortality of less than 40% was also observed for mosquitoes collected in polluted and non-polluted habitats. Mortality rates for permethrin ranged from 46 to 82% in Douala and from 57 to 90% in Yaoundé with the lowest mortality rates being recorded in cultivated areas in both cities. Deltamethrin exposure resulted in 86 to 94% mortality in Douala and 82 to 93% mortality in Yaoundé (Figure 5). The survival rate of mosquitoes exposed to DDT is significantly higher in Douala (81%) compare to Yaoundé (52%) (P < 0.0001), whereas survival to permethrin or deltamethrin did not differ significantly between the two sites (P > 0.29)

### *Kdr *genotyping

A total sample of 781 mosquitoes were genotyped for the 1014 *kdr *allele using the Hot oligonucleotide ligation assay of Lynd [26]. A large number (319, 40.8%) gave ambiguous results and these were therefore genotyped using the Taqman method [27]. A subset of the successful HOLA samples were also genotyped using the Taqman assay and a concordance rate of 95% (n = 40) was obtained. The L1014F allele was prevalent in all samples (Table 4). Only one specimen in Douala was found with the L1014S allele. The frequency of the *kdr *allele varied from 68.2% in Douala to 44.3% in Yaoundé and was significantly different (P < 0.0001). The *kdr *allele frequency also varied significantly according to breeding habitats (P < 0.0001). It was highest in cultivated areas followed by non-polluted sites of both Douala and Yaoundé (Table 5). Hardy Weinberg expectations were significantly rejected when samples from each city were considered belonging to single panmictic units (Douala: P = 0.002; Yaoundé P = 0.0003). When the pooled sample of each city was split according to breeding site origin, no significant deviation to Hardy Weinberg equilibrium was detected in Douala while in Yaoundé, the population from non-polluted sites did not conform to Hardy Weinberg expectations (Table 5).

**Table 4:** Distribution of the Kdr genotype within An. gambiae M and S forms in Douala and Yaoundé

	An. gambiae molecular forms		Genotypes					
		
		Kdrw/Kdrw	Kdrw/KdrE	Kdrw/S	S/S	Total	F(Kdr)	P(HW)
Douala	M form	228	1	170	62	460	0.68	P = 0.002
	S form	-	-	1	-	1		
Yaoundé	M form	79	0	124	115	318	0.44	P = 0.0003
	S form	-	-	2	-	2		
Total		307	1	296	177	781		

S, wild type; Kdrw, L1014F substitution « West Africa"; KdrE, L1014S substitution "East Africa", Kdrw/S, heterozygote; P(HW), goodness of fit to Hardy Weinberg Equilibrium (significant if P < 0.05).

**Table 5:** Distribution of Kdr genotypes in An. gambiae populations from Douala and Yaoundé according to the category of breeding habitat and level of susceptibility to insecticides

Parameters	Douala					Yaoundé					All	
	
	RR	RS	SS	F(Kdr)	P(HW)	RR	RS	SS	F(Kdr)	P(HW)	N	F (Kdr)
Breeding sites												
Polluted	35	44	22	0.56	0.31	10	36	39	0.33	0.81	186	0.45
Non polluted	101	95	33	0.65	0.26	50	85	73	0.44	0.01	437	0.55
Cultivated	92	31	7	0.83	0.06	19	5	2	0.83	0.13	156	0.83
Insecticides												
DDT 4%												
Deaths	2	7	6	0.37	-	2	5	30	0.12	-	52	0.19
Survivors	27	29	8	0.65	-	9	23	8	0.51	-	104	0.59
Permethrin 0.75%												
Deaths	48	68	24	0.58	-	2	25	23	0.29	-	190	0.51
Survivors	85	16	4	0.88	-	17	10	4	0.71	-	136	0.84
Deltamethrin 0.05%												
Deaths	37	34	13	0.64	-	29	30	18	0.57	-	161	0.61
Survivors	21	9	5	0.73	-	8	9	5	0.56	-	57	0.67

S, wild type; R, 1014F; RS, heterozygote; RR, homozygote resistant; P(HW), goodness of fit to Hardy Weinberg Equilibrium (significant if P < 0.05); F(kdr), frequency of the Kdr allele.

Comparisons of the *Kdr *allele frequency between dead and surviving mosquitoes after exposure to DDT, permethrin, and deltamethrin, revealed a significantly high *kdr *allele frequency in survivors to DDT and permethrin bioassay compare to non survivors. No significant difference was recorded with deltamethrin.

Odd ratio calculations to determine the strength of association between genotypes and phenotypes (dead or surviving) revealed that individuals carrying the *kdr *allele at both homozygote and heterozygote states were more resistant than the wild type to DDT. Only *kdr *homozygotes were resistant to permethrin. No association was detected between the presence of the *kdr *allele and resistance to deltamethrin (Table 6). A sample of 74 anopheline tested against bendiocarb were screened for the presence of the ace-1R mutation. No mosquito was found with this mutation.

**Table 6:** Strength of association between genotypes related to Kdr and the phenotype deaths or survivors of An. gambiae in Douala and Yaoundé.

	Insecticide	Comparison between genotypes		
		
		RR Vs. RS	RR Vs. SS	RS Vs. SS
Douala	DDT 4%	3.26 (NS)	10.12*	3.1 (NS)
	Permethrin 0.75%	7.5**	10.6**	1.4 (NS)
	Deltamethrin 0.05%	2.14 (NS)	1.48 (NS)	0.69 (NS)
Yaoundé	DDT 4%	0.98 (NS)	16.9**	17.2**
	Permethrin 0.75%	21.25**	48.9**	2.3 (NS)
	Deltamethrin 0.05%	0.92 (NS)	0.99 (NS)	1.08 (NS)
Both cities	DDT 4%	2.08 (NS)	20.25**	9.5**
	Permethrin 0.75%	7.29**	11.98**	1.26 (NS)
	Deltamethrin 0.05%	1.56 (NS)	1.36 (NS)	0.87 (NS)

S, wild type; R, 1014F; RR, homozygote; RS, heterozygote; NS, Non Significant; *P < 0.05; **P < 0.001

## Discussion

Oviposition sites were numerous in Douala and Yaoundé throughout the year. As in other major African cities, many of these sites were positive for *An. gambiae *larvae confirming the adaptation of malaria vectors to the urban environment [29-32]. Although not all larvae will complete their development to adults and not all adults will become infected with malaria parasites, the adaptation of malaria vectors to urban areas alongside the increasing number of gametocyte carriers migrating from rural to urban areas is of concern since it could significantly modify the epidemiology and transmission risks [3]. In Douala and Yaoundé, the majority of anopheline breeding places were small water collections exposed to sunshine, consistent with the known preference of *An. gambiae *for temporary sites [31,33]. However, anopheline larvae were also found in organically polluted sites and in large water bodies (pools drains or rivers) which could have contributed to the maintenance of anopheline all year round particularly during the dry season. Few significant differences of the physicochemical characteristics of breeding sites originating from rural and urban areas were recorded.

Important differences were recorded between breeding sites from the two cities. These differences could be associated to the infiltration of sea water far inland in Douala and to the poor management of wastes deriving from industrial or domestic activities. Although no strong correlation could be detected between vector distribution and the physicochemical properties of breeding sites in both cities, it is probable that several of the measured parameters directly or indirectly affect anopheline development or potentially susceptibility to insecticides and need to be assessed. Several activities taking place in the city were found to create suitable oviposition sites. In particular, the colonization of lowland areas for living or for the practice of agriculture, public works and domestic activities were among the important sources of breeding opportunities for anopheline larvae.

The predominance of *An. gambiae *M molecular form over the S form was consistent with recent findings in Cameroon confirming the adaptation of the M form to urban areas while the S form is restricted to rural areas (Kamdem *et al *[34]). High susceptibility of *An gambiae *to organophosphates and carbamates was detected and this was similar to previous observations across Cameroon [11,12,35]. However, resistance to carbamates (bendiocarb) was detected in cultivated areas in Yaoundé. This is the second time since 2007 that resistance to carbamates (carbosulfan) is reported [36]. These findings highlight the recent emergence of this resistance across Cameroon. Due to the absence of the ace-1^R ^alleles in all mosquitoes tested, metabolic resistance is certainly the primary resistance mechanisms to bendiocarb in the population of Yaoundé.

A high frequency of resistance to both DDT and permethrin was detected and this resistance was particularly prevalent in mosquitoes collected in cultivated areas suggesting a high selective pressure in these sites. Indeed numerous insecticides such as pyrethroids (cypermethrin, deltamethrin, Pyriforce^® ^(chlorpyrifos ethyl) and lambdacyhalothrin), carbamates (carbofuran), organophosphates (Dimethoate, Diazinon), and organochlorines (Endosulfan) are frequently used by farmers to fight against pests [13]. These data are consistent with previous findings across the continent confirming the large scale development of vector resistance and stressing the role of agricultural practices in the emergence of this resistance [37-40]. DDT and permethrin resistance was more prevalent in the current study than in earlier studies in Cameroon [11-13,41] and highlights the rapid evolution of this resistance which could have benefited from the extension of cultivated land surfaces.

Although urban agricultural land surfaces in Yaoundé were 8 to 10 fold larger than in Douala, this was not correlated with the frequency of the *kdr *allele which was present at significantly higher frequencies in Douala than Yaoundé (0.44 in Yaounde to 0.68 in Douala). These *kdr *frequencies are considerably higher than previously reported [42,41,12,13,36], and suggest a high selective pressure on mosquito populations in urban areas. When comparing *kdr *allele frequencies between types of breeding sites, the frequency of the mutation was much greater in the cultivated sites. The difference between sites could result from the high selection of larvae. Probably, exposition to high concentrations of insecticides in cultivated sites is selectively killing larvae carrying the wild type allele at the homozygous and heterozygous states. Further, the large fluctuation of the *kdr *allele frequency between categories of breeding habitats in Yaoundé, reflects a less extensive selective pressure in this city. Indeed in Yaoundé, the low permeability of the soil favors slow elimination of standing water collections and so perhaps attenuates the effect of xenobiotics on larvae, while in Douala the high permeability of the soil combined with the high evaporation of water, may expose larvae to high concentrations of pollutants. It is possible that exposition to high concentration of xenobiotics might have increased selection for resistance to insecticides in Douala. Several additional factors such as the increase used of impregnated bed nets particularly, LLIN in these cities could have favored the selection of resistant populations to DDT and permethrin and this need further assessment.

There was little difference in insecticide resistance or *kdr *allele frequency in mosquitoes collected from the polluted versus the non-polluted sites. Several studies on the adaptation of *An. gambiae *to urban sites have suggested that pollution plays an important role in selecting for increased tolerance to insecticides [32,29,43]. Gene expression analyses have suggested that insecticide detoxification and potentially cuticular thickness are both increased in mosquitoes emerging from polluted environments [44]. However, the initial data from Cameroon suggests that urban agriculture, rather than pollution, is the major factor driving resistance to insecticides.

## Conclusion

This study provided many important findings that should be considered for future malaria control initiatives. Vector control strategies cannot uniquely rely on the promotion of ITNs as it is the case nowadays in Cameroon. In the cities of Douala and Yaoundé, most if not all breeding sites can be identified and accessed for vector control. Therefore, prevention strategies combining larval control can now be a central feature for urban malaria control. With the perspective of large expansion of vector resistance in the close future, larval control through larviciding or environmental management could be central to the long-term goal of eradication of malaria transmission in these urban settings.

## Competing interests

The authors declare that they have no competing interests.

## Authors' contributions

Conceived and designed the study protocol: CAN, HR, CSW. Participated in field and laboratory analyses, BTF, BM, CN, SZ, PAA, CAN. Help with statistical analysis: CC. Interpreted, analysed data and wrote the paper: CAN. All authors read and approved the final version of the manuscript.

## References

[B1] OMS Statistiques sanitaires mondiales 2009 1. Indicateur état sanitaire. 2.Santé mondiale. 3.Services santé - statistique. 4.Mortalité. 5.Morbidité 6.Espérance vie. 7.Démographie. 8.Statistique. I.Organisation mondiale de la Santé. ISBN 978 92 4 256381 8 (NLM classification: WA 900.1) 2009 1 49

[B2] Trape J Lefebvre-Zante E Legros FGN Bouganali H Druilhe P Salem G Vector density gradients and the epidemiology of urban malaria in Dakar, Senegal Am J Trop Med Hyg 1992 47 181 189 1354414 10.4269/ajtmh.1992.47.181

[B3] Keiser J Utzinger J Caldas de Castro M Smith T Tanner M Singer B Urbanization in sub-saharan Africa and implication for malaria control Am J Trop Med Hyg 2004 71 118 127 15331827

[B4] BUCREP Troisième recencement générale de la population et de l'habitat. Third general population and housing census Cameroun 2010 Rapport de présentation des résulstats définitifs République du Cameroun 1 6521641103

[B5] Nimpaye H M Van Der Kolk Fontenille D Boudin C Le paludisme urbain à Yaoundé (Cameroun) en 2000. Etude entomologique dans le quartier central «Dakar» Bull Liais Doc OCEAC 2001 34 11 14

[B6] Quakyi I Leke R Befidi-Mengue R Tsafack M Bomba-Nkolo D Manga L Tchinda V Njeungue E Kouontchou S Fogako J Nyonglema P Harun LT Djokam R Sama G Eno A Megnekou R Metenou S Ndoutse L Same-Ekobo A Alake G Meli J Ngu J Tietche F Lohoue J Mvondo JL Wansi E Leke R Folefack A Bigoga J Bomba-Nkolo C Titanji V Walker-Abbey A Hickey MA Johnson AH Taylor DW The epidemiology of Plasmodium falciparum malaria in two Cameroonian villages: Simbok and Etoa Am J Trop Med Hyg 2000 63 222 230 11421368

[B7] Peyrefitte C Rousset D Pastorino B Pouillot R Bessaud M Tock F Mansaray H Merle O Pascual A Paupy C Vessiere A Imbert P Tchendjou P Durand J Tolou H Grandadam M Chikungunya virus, Cameroon, 2006 Emerg Infect Dis 2007 13 768 771 17553262 10.3201/eid1305.061500 PMC2738435

[B8] Kamgang B HJ Boisier P Njiokou F Hervé JP Simard F Paupy C Geographic and ecological distribution of the dengue and chikungunya virus vectors *Aedes aegypti *and *Aedes albopictus *in three major Cameroonian towns Med Vet Entomol 2010 24 132 141 10.1111/j.1365-2915.2010.00869.x 20408956

[B9] Antonio-Nkondjio C Awono-Ambene H Toto J Meunier J Zebaze-Kemleu S Nyambam R Wondji C Tchuinkam T Fontenille D High malaria transmission intensity in sub-urban area of Yaounde: the capital city of Cameroon J Med Entomol 2002 39 350 355 10.1603/0022-2585-39.2.350 11931035

[B10] Minsanté Rapport du Programme National de lutte contre le paludisme du Cameroun 2010 Rapport sur l'évolution des cas d'accès palustres dans les districts de santé de la ville de Yaoundé 35

[B11] Etang J Manga L Chandre F Guillet P Fondjo E Mimpfoundi R Toto J Fontenille D Insecticide susceptibility status of Anopheles gambiae s.l. (Diptera: Culicidae) in the Republic of Cameroon J Med Entomol 2003 40 491 497 10.1603/0022-2585-40.4.491 14680116

[B12] Ndjemai H Patchoke S Atangana J Etang J Simard F Bilong Bilong C Reimer L Cornel A Lanzaro C Fondjo E The distribution of insecticide resistance in *Anopheles gambiae *s.l populations from Cameroon: an update Trans R Soc Trop Med Hyg 2008 10.1016/j.trstmh.2008.11.018 19155034

[B13] Nwane P Etang J Chouaibou M Toto J Kerah-Hinzoumbe C Mimpfoundi R Awono-Ambene H Simard F Trends in DDT and pyrethroid resistance in *Anopheles gambiae *s.s. populations from urban and agro-industrial settings in southern Cameroon BMC Infect Dis 2009 9 163 10.1186/1471-2334-9-163 19793389 PMC2764715

[B14] Girod R Salvan M Denys J Lutte contre la réintroductiondu paludisme à la Réunion Cah Sante AUPELF UREF 1995 5 401 405 8784548

[B15] Fillinger U Kannady K William G Vanek M Dongus S Nyika D Geissbuhler Y Chaki P Govella N Mathenge EM Singer BH Mshinda H Tanner M Mtasiwa D de Castro MC Killeen GF A tool box for operational mosquito larval control: preliminary results and early lessons from the Urban Malaria Control Programme in Dar es Salaam, Tanzania Malar J 2008 7 20 10.1186/1475-2875-7-20 18218148 PMC2259364

[B16] Shililu J Tewolde G Brantly E Githure J Mbogo C Beier J Fusco R Novak B Efficacy of *Bacillus thuringiensis israelensis*, *Bacillus sphaericus *and temephos for managing Anopheles larvae in Eritrea J Am Mosq Control Assoc 2003 19 251 258 14524547

[B17] de Castro M Kannady K Singer B Mtasiwa D Mshinda H Tanner M Geissbuhler Y Lindsay S Fillinger U Killeen G Reduction in malaria prevalence in Dar es Salaam, Tanzania following vector control with microbial larvicides PLoS Med 2007

[B18] WHO Manual on practical entomology in malaria. Part II 1975 World Health Organization, Genava 45

[B19] Gillies M Coetzee M A supplement to the Anophelinae of Africa south of the Sahara (Afrotropical region) Pub South Afr Inst Med Res 1987 55 143

[B20] Edwards F 1941 London: British Museum (Natural History)

[B21] Fanello C Petrarca V della Torre A Santolamazza F Dolo G Coulibaly M Alloueche A Curtis C Toure Y Coluzzi M The pyrethroid knock-down resistance gene in the *Anopheles gambiae *complex in Mali and further indication of incipient speciation within *Anopheles gambiae *s.s Insect Mol Biol 2003 12 241 245 10.1046/j.1365-2583.2003.00407.x 12752657

[B22] Livak K Organization and mapping of a sequence on the *Drosophila melanogaster *X and Y chromosomes that is transcribed during spermatogenesis Genetics 1984 107 611 634 6430749 10.1093/genetics/107.4.611 PMC1202380

[B23] Cornel A Collins F PCR of the ribosomal DNA intergenic spacer regions as a methods for identifying mosquitoes in the *Anopheles gambiae *complex Methods mol Biol 1996 56 321 332 10.1385/0-89603-323-6:321 8751368

[B24] WHO Test procedures for insecticide resistance monitoring in malaria Vectors. Bio-efficacy and Persistence of insecticides on treated surfaces 1998 WHO/MAL/98,12 Report of the WHO Informal Consultation, Geneva 43

[B25] Abbott WS A method of computing the effectiveness of an insecticide J Ecol Entomol 1925 18 265 267 3333059

[B26] Lynd A Ranson H McCall P Randle N Black W Walker E Donnelly M A simplified high-throughput method for pyrethroid knock-down resistance (kdr) detection in *Anopheles gambiae* Malar J 2005 4 16 10.1186/1475-2875-4-16 15766386 PMC555548

[B27] Bass C Nikou D Donnelly M Williamson M Ranson H Ball A Vontas J Field L Detection of knockdown resistance (kdr) mutations in *Anopheles gambiae: *A comparison of two new high-throughput assays with existing methods Malar J 2007 6 111 10.1186/1475-2875-6-111 17697325 PMC1971715

[B28] Raymond M Rousset F Genepop (version-1.2)-Population-genetics software for exact tests and ecumenicism J Hered 1995 86 248 249

[B29] Awolola T Oduola A Obansa J Chukwurar N Unyimadu J *Anopheles gambiae *s.s. breeding in polluted water bodies in urban Lagos, southwestern Nigeria J Vector Borne Dis 2007 44 241 244 18092529

[B30] Afrane Y Klinkenberg E Drechsel P Owusu-Daaku K Garms R Kruppa T Does irrigated urban agriculture influence the transmission of malaria in the city of Kumasi, Ghana? Acta Trop 2004 89 125 134 10.1016/j.actatropica.2003.06.001 14732235

[B31] Sattler M Mtasiwa D Kiama M Premji Z Tanner M Killeen G Lengeler C Habitat characterization and spatial distribution of Anopheles sp. mosquito larvae in Dar es Salaam (Tanzania) during an extended dry period Malar J 2005 4 4 10.1186/1475-2875-4-4 15649333 PMC546229

[B32] Djouaka R Bakare A Bankole H Doannio J Coulibaly O Kossou H Tamo M Basene H Popoola O Akogbeto M Does the spillage of petroleum products in Anopheles breeding sites have an impact on the pyrethroid resistance? Malar J 2007 6 159 10.1186/1475-2875-6-159 18053173 PMC2222605

[B33] Edillo F Toure Y Lanzaro G Dolo G Taylor C Spatial and habitat distribution of *Anopheles gambiae *and *Anopheles arabiensis *(Diptera: Culicidae) in Banambani village, Mali J Med Entomol 2002 39 1 70 77 10.1603/0022-2585-39.1.70 11931274

[B34] Simard F Ayala D Kamdem G Pombi M Etouna J Ose K Fotsing J Fontenille D Besansky N Costantini C Ecological niche partitioning between *Anopheles gambiae *molecular forms in Cameroon: the ecological side of speciation BMC Ecology 2009 9 17 10.1186/1472-6785-9-17 19460146 PMC2698860

[B35] Antonio-Nkondjio C Atangana J Ndo C Awono-Ambene P Fondjo E Fontenille D Simard F Malaria transmission and rice cultivation in Lagdo, northern Cameroon Trans R Soc Trop Med Hyg 2008 102 352 359 10.1016/j.trstmh.2007.12.010 18295810

[B36] Bigoga JD Manga L Titanji VPK Etang J Coetzee M Leke RGF Susceptibility of *Anopheles gambiae *Giles (Diptera: Culicidae) to pyrethroids, DDT and carbosulfan in coastal Cameroon African Entomology 2007 15 1 1 7 10.4001/1021-3589-15.1.1

[B37] Ranson H Abdalla H Badolo A Guelbeogo W Kerah-Hinzoumbe C Yangalbe-Kalnone E Sagnon N Simard F Coetzee M Insecticide resistance in Anopheles gambiae: data from the first year of a multi-country study highlight the extent of the problem Malar J 2009 8 299 10.1186/1475-2875-8-299 20015411 PMC2804687

[B38] Yadouleton A Asidi A Djouaka R Braima J Agossou C Akogbeto M Development of vegetable farming: a cause of the emergence of insecticide resistance in populations of *Anopheles gambiae *in urban areas of Benin Malar J 2009 8 103 10.1186/1475-2875-8-103 19442297 PMC2686728

[B39] Corbel V N'Guessan R Brengues C Chandre F Djogbenou L Martin T Akogbeto M Hougard J Rowland M Multiple insecticide resistance mechanisms in *Anopheles gambiae *and *Culex quinquefasciatus *from Benin, West Africa Acta Trop 2007 101 207 216 10.1016/j.actatropica.2007.01.005 17359927

[B40] Diabate A Baldet T Chandre F Akogbeto M Darriet F Brengues C Guiguemde T Guillet P Hemingway J Hougard J The role of agricultural use of insecticides in resistance to pyrethroids in *Anopheles gambiae *s.l. in Burkina Faso Am J Trop Med Hyg 2002 67 617 622 12518852 10.4269/ajtmh.2002.67.617

[B41] Reimer L Fondjo E Patchoke S Diallo B Lee Y Arash N Ndjemai H Atangana J Traore S Lanzaro G Cornel A Relationship between kdr mutation and resistance to pyrethroid and DDT insecticides in natural populations of *Anopheles gambiae* J Med Entomol 2008 45 2 260 266 10.1603/0022-2585(2008)45[260:RBKMAR]2.0.CO;2 18402142

[B42] Etang J Fondjo E Chandre F Brengues C Nwane P Chouaubou M Ndjemai H Simard F First report of knockdown mutations in the malaria vector *Anopheles gambiae *from Cameroon Am J Trop Med Hyg 2006 74 795 797 16687682

[B43] Awolola T Oduola O Strode C Koekemoer L Brooke B Ranson H Evidence of multiple pyrethroid resistance mechanisms in the malaria vector *Anopheles gambiae *sensu stricto from Nigeria Trans R Soc Trop Med Hyg 2009 103 1139 1145 10.1016/j.trstmh.2008.08.021 18829056

[B44] Djouaka R Bakare A Coulibaly O Akogbeto M Ranson H Hemingway J Strode C Expression of the cytochrome P450s, CYP6P3 and CYP6M2 are significantly elevated in multiple pyrethroid resistant populations of *Anopheles gambiae *s.s. from Southern Benin and Nigeria BMC Genomics 2008 9 538 10.1186/1471-2164-9-538 19014539 PMC2588609

